# Effect of Pethidine Injection on the Duration of Labor and Pregnancy Outcomes: A Retrospective Cohort Study

**DOI:** 10.3390/medicina60010143

**Published:** 2024-01-12

**Authors:** Eun Byeol Cho, Hyun Joo Chae, Jung Min Ryu, Hyo Jin Lee, Seong Yeon Hong, Jin Young Bae

**Affiliations:** Department of Obstetrics and Gynecology, School of Medicine, Catholic University of Daegu, Daegu 42472, Republic of Korea; brownie217@naver.com (E.B.C.); chj3706@naver.com (H.J.C.); medgirl87@naver.com (J.M.R.); babydino04@naver.com (H.J.L.); magu815@cu.ac.kr (S.Y.H.)

**Keywords:** active phase, labor duration, pethidine, pregnancy outcome

## Abstract

*Background and Objectives*: Long and ineffective labor causes hardships for mothers and doctors and increases the rate of cesarean sections and medical comorbidities. Several factors contribute to effective and less painful labor, including maternal age, parity, fetal characteristics, and the medications or procedures that obstetricians use for labor. We aimed to study the factors that affect labor duration and identify those that make labor more effective. *Materials and Methods*: This retrospective study included 141 patients who underwent normal vaginal deliveries at the Daegu Catholic University Medical Center between April 2013 and April 2022. Among the 141 patients, 44 received pethidine intravenously, 88 received oxytocin intravenously, and 64 received epidural anesthesia. The duration of the active phase and second stage of labor were recorded according to the findings of a manual examination of the cervix and continuous external electronic monitoring. We analyzed maternal and neonatal medical records and performed binomial logistic regression to identify the factors associated with a shorter active phase of labor. The clinical outcomes in mothers and neonates were also evaluated. *Results*: Among the various clinical factors, multiparity (odds ratio of parity 0.325) and the use of pethidine (odds ratio 2.906) were significantly associated with shortening the active phase of labor to less than 60 min. The use of epidural anesthesia or oxytocin was not significantly associated with reducing the active phase of labor. When patients were divided into two groups based on whether a pethidine injection had been used during labor, the duration of the active phase was shorter in the pethidine injection group than in the control group for both nulliparas and multiparas. No significant differences in the duration of the second stage of labor were observed between the pethidine injection and control groups. There were no significant differences in pregnancy outcomes, including the need for mechanical ventilation of neonates, Apgar scores, neonatal intensive care unit admissions, number of precipitous deliveries, maternal adverse side effects of drugs, or duration of maternal hospitalization between the two groups. *Conclusions*: Pethidine can be safely administered to women during labor to help reduce the duration of the active phase by promoting dilatation of the cervix and preventing complications that may result from prolonged labor. Pethidine may be helpful, especially for those who cannot receive epidural anesthesia or who cannot afford it. However, large-scale randomized controlled studies are required to evaluate the efficacy and safety of this drug during labor. Furthermore, it would be helpful if various studies were conducted depending on the timing of administration and indications for delivery.

## 1. Introduction

Labor pains are characterized by powerful and painful uterine contractions. The mother’s cervix dilates and thins, allowing the fetus to descend through the birth canal. The onset of labor pains is the result of complex biochemical changes in the uterus and cervix [1]. Labor progress is greatly influenced by the condition of the cervix. Cervical dilatation and effacement are influenced by various factors, including maternal age and parity, uterine contraction frequency and intensity, cervical compliance, fetal head position and descent, fetal size, and medication administration [2,3]. Traditionally, the active phase of labor was believed to begin at 4 cm cervical dilatation, after which labor is considered to progress more rapidly according to Friedman labor curve [4]. However, Zhang et al. presented a new indication for the onset of active labor at 6 cm cervical dilatation. In their large study using the medical records of 19 hospitals, they reported that cervical dilatation showed acceleration after 6 cm of dilatation, and the progress from 4 to 6 cm was slower than previously perceived. The average duration of the active phase has been reported as 2.1 h in nulliparous women and 1.5 h in multiparous women [5,6]. The results of their study formed the basis of new guidelines for determining the arrest of labor and the need for a cesarean section [7].

When the dilatation of the cervix is too slow or stops, it is considered an active-phase disorder (protraction or arrest), and subsequent pharmacological augmentation, especially intravenous oxytocin administration, is usually performed [8]. Long and ineffective labor causes hardships for many mothers and increases the rate of cesarean sections, maternal morbidity, hospital stays, and medical costs. In addition, extreme labor pain can cause pain catastrophization, which can have a negative impact on the mother’s physical and mental recovery after delivery [9]. To relieve labor pain, epidural anesthesia or the administration of painkillers are considered, and relaxation techniques, such as sacral massage or aromatherapy, may be used to relieve labor pains. Such medical interventions can affect the progress of labor [10,11,12].

Therefore, we aimed to evaluate the various clinical factors that affect the labor course and identify the factors that make labor more effective and less painful.

## 2. Materials and Methods

This retrospective study included pregnant women who underwent normal vaginal deliveries at the Daegu Catholic University Medical Center between April 2013 and April 2022. The inclusion criteria were as follows: patients were women aged from 16 to 45 years with vaginal deliveries, singleton pregnancies with vertex presentation, and gestational age at delivery ranging from 37 + 0 weeks to 41 + 1 weeks. The exclusion criteria were as follows: patients with medical problems (major organ disorders such as diabetes, hypertension, cardio-pulmonary disease, musculoskeletal disorders that can affect the progress of labor, or systemic infection), abnormal external fetal electronic monitoring findings, cephalopelvic disproportion, previous history of uterine surgery, multiple gestations, estimated fetal weight greater than 4000 g, and obstetric complications such as preeclampsia, fetal anomalies, preterm labor, and fetal growth restriction. A total of 141 patients met all the above conditions and were surveyed.

The course of labor was monitored in 141 patients, and continuous external electronic monitoring was applied to examine maternal uterine contractions and fetal heart rate. Intravenous oxytocin was used to augment labor when the uterine contractions of the patients were insufficient. Inappropriate uterine contractions were considered when the intensity of uterine contractions was less than 200 Montevideo units on intrapartum external electronic monitoring. Oxytocin (5 IU in 500 mL of normal saline) was administered at a rate of 2.5 mIU/minute and increased by 2.5 mIU/minute every 20 min intravenously to achieve regular contractions (more than 200 Montevideo units). When cervical dilatation was 3–5 cm, 44 patients received a 25 mg bolus injection of pethidine intravenously, and 64 patients received an intervention of epidural anesthesia.

The reasons for receiving pethidine were as follows: the mother’s financial request, gestational thrombocytopenia (platelet count under 100,000/uL), the mother did not want to receive an epidural, or the mother refused a spinal procedure due to a pre-existing back condition. After administering pethidine, the mother’s blood pressure was checked every 10 min, and she was also checked for nausea, dizziness, difficulty breathing, or other symptoms suggesting side effects of pethidine.

### 2.1. Method of Epidural Anesthesia in Labor

The patient is positioned on the left lateral position, with the back curved to widen the spaces between the vertebrae. After carefully disinfecting the mother’s back with antiseptic, the patient is numbed with 3–5 mL of 1% lidocaine injection. A Tuohy epidural needle is inserted between lumbar vertebrae 3 and 4 or 4 and 5 intervertebral space into the epidural space. The needle is guided by feeling the loss of resistance as it passes through different tissues. A thin, flexible catheter is threaded through the needle and is placed roughly 3–5 cm into the epidural space. The needle is then removed, leaving the catheter in place. The catheter is taped to the skin and connected to a pump that delivers a 10 mL mixture of 0.175% ropivacaine. Epidural solutions are administered by bolus, and additional bolus solution is given when the patient complains of pain with same regimen via catheter. Then, changes in pain are observed while closely monitoring the mother’s blood pressure during labor. Epidural anesthesia catheter is removed one hour after delivery.

General physical and obstetric examinations were performed on all patients. External electronic monitoring and manual cervical dilation measurements were performed every hour to determine labor progression. When a mother complained of severe pain or a sinking feeling, additional examination was performed immediately. When necessary, vaginal delivery was performed in accordance with the routine protocol of our hospital, including episiotomy. There were no mothers who had instrumental deliveries.

The durations of active phase and second stage of labor were recorded according to the findings of manual examination of cervix and continuous external electronic monitoring. The duration of the active phase of labor was defined as the time (minutes) from 6 cm cervical dilatation to full cervical dilatation [5,6]. The duration of the second stage of labor was defined as the time (minutes) from full cervical dilatation to delivery of the neonate. The following clinical outcomes of mothers and newborns were recorded and analyzed: mother (maternal age; body mass index; parity; gestational age; reason for induction; use of pethidine, oxytocin, or epidural anesthesia; hospital stay; and any other complications) and neonate (birth weight, use of mechanical ventilation, 1 min and 5 min Apgar scores, and neonatal intensive care unit admission (NICU) and the reason).

### 2.2. Statistical Analysis

Data were analyzed using SPSS statistical software (V25.0; IBM, Armonk, NY, USA). Variables were analyzed using binomial logistic regression. The duration of labor and maternal–neonatal outcomes were analyzed using independent sample t-tests and chi-square tests. *p*-values were obtained using two-tailed tests; *p* < 0.05 was considered statistically significant. This retrospective study was approved by the Ethics Committee of Daegu Catholic University Medical Center (approval number CR-23-104). As this study involved retrospective data collection, informed consent was not required.

## 3. Results

Maternal and neonatal characteristics of the participants are presented in Table 1. Among the 141 patients, 44 received pethidine intravenously, 88 received oxytocin intravenously, and 64 received epidural anesthesia.

We analyzed various clinical factors that affected the labor course of the participants using binomial logistic regression. According to Zhang et al., the average time of the active phase in multiparous women who underwent spontaneous labor is 1.5 h [6]; therefore, we sought to identify the clinical factors associated with reducing the active phase to less than 60 min. The factors related to an active phase of less than 60 min were multiparity (*p* = 0.014) and the use of pethidine (*p* = 0.025); the use of pethidine showed a higher correlation with a shorter duration of the active phase (odds ratio 2.906) (Table 2). The active phase was prolonged in patients who used epidural anesthesia, as reported by Zhang et al. (odds ratio 0.422) [6].

Based on these results, we divided the patients into two groups: those who used pethidine and those who did not use pethidine (control group) and analyzed the duration of the active phase and second stage. The duration of the active phase was significantly shorter in the pethidine injection group than in the control group (56.89 ± 38.53 min vs. 89.89 ± 73.53 min, *p* < 0.001) (Table 3). The pethidine injection group had a significantly shorter active phase duration in both nulliparous (68.46 ± 42.63 min vs. 108.80 ± 90.51 min, *p* = 0.018) and multiparous (40.17 ± 24.13 min vs. 76.61 ± 55.93 min, *p* < 0.001) women. For the duration of the second stage, no significant difference was observed between the pethidine injection and control groups (17.05 ± 19.99 min vs. 20.24 ± 18.33 min).

Parturients who received oxytocin included 29 mothers (66%) of the pethidine group and 59 mothers (61%) of the control group. Our subanalysis showed no significant statistical differences in oxytocin use between the pethidine and control groups (*p* = 0.564). Of the control group, 57 (59%) received epidural anesthesia.

The neonatal and maternal outcomes are presented in Table 4. No significant difference was noted in neonatal outcomes between the pethidine injection and control groups in terms of mechanical ventilation and cases with 1 min and 5 min Apgar scores of 7 or lower. The NICU admission rate was higher in the pethidine injection group; however, the difference was not statistically significant. Among the NICU admissions, the rate of admission due to respiratory failure, which is related to the effect of pethidine, was not significantly different between the groups. Other reasons for NICU admission included the need to evaluate children of mothers with prolonged ruptured membranes, maternal systemic lupus erythematosus, gestational diabetes, and neonates with skull fractures.

Regarding maternal outcomes, no significant differences were observed between the pethidine injection and control groups in terms of delivery or the duration of hospitalization. No other complications of the pethidine injection, including nausea, vomiting, or respiratory distress, occurred in any patient who received pethidine. In addition, since pethidine has the effect of shortening the active period of labor, we investigated whether it causes complications of precipitous delivery. Precipitous delivery means that the fetus is delivered within 3 h of the start of labor. In the group receiving pethidine, only one precipitous delivery was observed, and there was no significant difference compared to the control group (*p* = 0.582).

## 4. Discussion

The findings of our study suggest that pethidine administration during labor reduces the duration of the active phase of labor and has good maternal–neonatal outcomes. Pethidine, also known as meperidine, is inexpensive and one of the most commonly used drugs used during labor to reduce pain and facilitate vaginal delivery [10,11,13]. There are various clinical indications for its use, including financial reasons, staff constraints, and failed epidural anesthesia due to various reasons. Based on these observations, several physicians have reported that pethidine administration aids in pain relief and decreases labor duration [14,15].

It has been hypothesized that pethidine affects labor duration by altering uterine contractions and cervical ripening, and various reports have described the mechanisms underlying these effects. Mixed uterine contraction outcomes have been reported. Several studies have shown that intravenous injections of pethidine before labor are likely to increase uterine contractions [16,17]. An in vitro study in rats showed that pethidine administration inhibited the contractility of pregnant rat myometrium [18]. Collagen, the major structural protein of the cervix, undergoes structural changes during the process of labor, resulting in changes in tissue flexibility [19]. During cervical ripening, a pethidine-induced increase in urokinase activity converts plasminogen into active plasmin. Upon plasmin formation, latent pro-collagenase is converted into active collagenase, resulting in the rapid cleavage of cervical collagen and causing cervical changes [20]. This may explain the effects of pethidine administration on labor progression.

Safely reducing labor time is important because long and painful labor exhausts the mother and increases the rate of cesarean sections. As a result, medical costs increase, and maternal complications may also increase. Additionally, frequent internal manual examinations by medical personnel during long labor times can cause chorioamnionitis [21]. Epidural anesthesia is well known as a medical treatment that reduces pain during labor for many mothers. However, several studies and guidelines have shown that although epidural anesthesia can reduce labor pains, it is not effective in shortening the duration of labor. Rather, epidural anesthesia may further prolong labor duration [6,7]. In addition, epidural anesthesia has several contraindications, including the following: maternal hypotension, patients who cannot communicate, coagulopathy, untreated bacteremia, a poor maternal back condition, a skin or soft tissue infection, and anticoagulant therapy. And the success rate may vary depending on the anesthesiologist’s skill level and maternal obesity. Also, the dosage of the drug is important. Higher doses of anesthetic drugs can cause maternal sensory and motor abnormalities, preventing the mother from feeling pain and exerting force [10].

Our study found that among several clinical factors thought to be related to the pace of labor progress, only two factors were able to significantly reduce the active phase labor duration to less than 60 min without interference from other factors in binomial logistic regression: multi-parity (odd ratio of parity: 0.325) and the use of pethidine (odd ratio: 2.906). It is well known that parity is related to labor duration [22], and, of course, parity is not applicable for medical intervention. The results also show that a pethidine injection consistently shortens the active phase labor duration across both primiparous and multiparous mothers in the following analysis, and that is the most important result of this study. Additionally, other studies have reported that gestational age, newborn weight, and the use of oxytocin affect delivery time, but our study did not show a statistically significant effect [3,22]. This may be a result of the insufficient number of participants included in this study.

Some studies have investigated the relationship between pethidine injections and labor duration. However, these findings are inconsistent, and no clear associations have been reported. In one study, no significant difference was observed between the pethidine injection and control groups in terms of labor duration; however, this study was limited to patients with uterine dystocia [23]. Another study conducted in Turkey showed that pethidine administration was associated with a shorter active phase of labor in primiparous women; however, this association was not observed in multiparous women [24].

We found that pethidine injections shortened the duration of the active phase in both primiparas and multiparas. However, the administration of pethidine was not effective in shortening the second stage. The second stage is the period after the cervix has fully dilated, and the effect of pethidine on the cervix no longer affects the progress of labor. These results demonstrate the effect of pethidine on cervical change.

According to the results of this study, pethidine is a drug that reduces the duration and pain of labor. However, all opioids may have theoretical side effects such as respiratory depression, nausea, vomiting, and sedation. Opioids may also influence fetal heart rate variability by crossing the placenta via passive diffusion in large amounts [11]. Therefore, we conducted an analysis of the safety outcomes for the group that used pethidine and the group that did not. There were no significant side effects in mothers and neonates when using pethidine in labor. We found no significant differences in the 1 min and 5 min Apgar scores in neonates. Our study also showed that pethidine injections did not increase the need for mechanical ventilation for respiratory failure in neonates. The reasons for NICU admission other than respiratory failure included the need to evaluate the effects of prolonged rupture of membranes (over 18 h), patients with systemic lupus erythematosus, gestational diabetes, and neonatal skull fracture. No maternal complications, including nausea, vomiting, or respiratory distress, were associated with pethidine use.

Previous studies on pethidine injections during labor have reported inconsistent but mostly safe neonatal outcomes. One study found that the pH of umbilical cord artery samples was lower in the pethidine group than in the control group; however, the difference was not statistically significant [23]. A Polish study showed that a pethidine injection during labor did not affect oxygen saturation, heart rate, or blood pressure in infants during the first 24 h of life [12]. A study conducted in Iran showed no significant difference in 5 min Apgar scores between the pethidine and control groups; however, 1 min Apgar scores were significantly higher in the pethidine group [25]. Another study showed no significant differences in 1 min and 5 min Apgar scores between the pethidine and control groups [24].

Most women experience satisfactory pain relief during labor after a pethidine injection [26,27,28]. In a Cochrane review of the use of parenteral opioids during labor, there was no clear difference between the pethidine and placebo groups in terms of nausea or vomiting [11]. However, because pethidine can shorten the duration of labor, there is a theoretical possibility that it could precipitate labor and its associated complications. Our data showed no significant differences in the rate of precipitous labor or the duration of hospitalization between the pethidine injection and control groups.

Considering our findings, we suggest that pethidine can be safely administered to women during labor to help reduce the duration of the active phase and prevent complications that may result from prolonged labor. We have observed the effect of administering pethidine when the mother’s cervical dilatation is 3–5 cm to shorten labor duration; therefore, we believe that the appropriate time to administer pethidine is at the beginning of the active phase. However, it would be more helpful if there were large-scale studies on the timing of pethidine administration at various times. In addition, research is needed to determine when it is appropriate to administer it to mothers who do not have natural labor at all and who require labor induction according to obstetric indications.

In conclusion, pethidine may be helpful, especially for patients for whom epidural anesthesia cannot be administered or who cannot afford it.

### Study Limitations

As this was a retrospective study, the size of the pethidine injection group was small. We expect that this limitation will be overcome in future large-scale studies. In our labor ward, cervical dilatation was monitored every two hours during the latent phase and every hour during the active phase or whenever there was a change in symptoms. A limitation of this study is the lack of objective measurements. However, I believe this is a limitation that most obstetric studies dealing with the progress of labor have [29]. Considering that cervical dilatation was assessed by different physicians, it could only be characterized subjectively.

## 5. Conclusions

Our findings suggest that pethidine administration is safe for shortening the duration of the active phase of labor. However, a large-scale, randomized, controlled study is required to confirm the efficacy and safety of pethidine during labor. Additionally, it would be helpful if various studies were conducted depending on the timing of administration and indications for delivery.

## Figures and Tables

**Table 1:** Maternal and neonatal characteristics.

	Overall Group (n = 141)
Maternal age (years, mean ± SD)	33.16 ± 5.06
Parity	
Nullipara	66
Multipara	75
Maternal BMI (kg/m^2^, mean ± SD)	21.10 ± 2.82
Gestational age (weeks, mean ± SD)	39.2 ± 0.9
Reason for induction	
Oligohydramnios	3
Post-term pregnancy	36
PROM	39
Pethidine injection	44
Oxytocin injection	88
Epidural anesthesia	64
Neonatal birth weight (grams, mean ± SD)	3182.41 ± 404.57

BMI, body mass index; PROM, premature rupture of membrane; SD, standard deviation.

**Table 2:** Factors associated with shorter active phase of labor (<60 min)—binomial logistic regression.

	Odd Ratio	95% CI	*p*-Value
Maternal age	0.932	0.855–1.017	0.112
Gestational age	0.983	0.924–1.046	0.598
Parity	0.325	0.133–0.795	0.014
Maternal BMI	0.936	0.806–1.086	0.381
Neonatal birth weight	1.001	1.000–1.002	0.072
Use of pethidine	2.906	1.140–7.405	0.025
Use of oxytocin	1.735	0.777–3.874	0.179
Use of epidural anesthesia	0.422	0.184–0.967	0.041

CI, confidence interval; BMI, body mass index.

**Table 3:** Duration of the active phase and second stage of labor.

	**Pethidine Injection Group** **(n = 44)**	**Control Group** **(n = 97)**	***p*** **-Value**
Active phase (min)	56.89 ± 38.53	89.89 ± 73.53	*p* < 0.001
Nullipara (n = 66)	68.46 ± 42.63 (n = 26)	108.80 ± 90.51 (n = 40)	*p =* 0.018
Multipara (n = 75)	40.17 ± 24.13 (n = 18)	76.61 ± 55.93 (n = 57)	*p <* 0.001
Second stage (min)	17.05 ± 19.99	20.24 ± 18.33	*p* = 0.353
Nullipara (n = 66)	22.12 ± 24.26 (n = 26)	28.50 ± 22.26 (n = 40)	*p =* 0.276
Multipara (n = 75)	9.72 ± 6.99 (n = 18)	14.44 ± 12.16 (n = 57)	*p =* 0.123

min, minutes. Data are presented as mean ± SD.

**Table 4:** Neonatal and maternal outcomes.

	Pethidine Injection Group (n = 44)	Control Group (n = 97)	*p*-Value
Neonatal Outcomes			
Mechanical ventilation	2 (4.5)	4 (4.1)	*p* = 0.908
Apgar score (1 min)			
≤7	4 (9.1)	8 (8.2)	*p* = 0.533
7>	41 (93.2)	89 (91.8)	
Apgar score (5 min)			
≤7	0 (0)	1 (1.0)	*p* = 0.688
7>	44 (100)	96 (98.9)	
NICU admission	11 (25)	12 (12)	*p* = 0.060
* Due to respiratory failure	6 (13.6)	5 (5.1)	*p* = 0.855
Maternal Outcomes			
Precipitous delivery	1 (2.2)	4 (4.1)	*p =* 0.582
Hospitalization period (days, mean ± SD)	3.55 ± 0.76	3.47 ± 0.66	*p =* 0.574
Side effects (nausea, vomiting, respiratory distress)	0 (0)	0 (0)	

Values are presented as number (%). min, minutes; NICU, neonatal intensive care unit; SD, standard deviation. * Other reasons for NICU admission: evaluation due to long period of ruptured membrane, mother with systemic lupus erythematosus, gestational diabetes, or neonatal skull fracture.

## Data Availability

The data presented in this study are available on request from the corresponding author. The data are not publicly available due to it contains the patient’s personal information.

## References

[1] Liao J.B., Buhimschi C.S., Norwitz E.R. (2005). Normal labor: Mechanism and duration. Obstet. Gynecol. Clin. N. Am..

[2] Hamilton E.F., Warrick P.A., Collins K., Smith S., Garite T.J. (2016). Assessing first-stage labor progression and its relationship to complications. Am. J. Obstet. Gynecol..

[3] Chen H., Cao L., Cao W., Wang H., Zhu C., Zhou R. (2018). Factors affecting labor duration in Chinese pregnant women. Medicine.

[4] Friedman E.A., Kroll B.H. (1969). Computer analysis of labour progression. J. Obstet. Gynaecol. Br. Commonw..

[5] Caughey A.B. (2020). Is Zhang the new Friedman: How should we evaluate the first stage of labor?. Semin. Perinatol..

[6] Zhang J., Landy H.J., Ware Branch D., Burkman R., Haberman S., Gregory K.D., Hatjis C.G., Ramirez M.M., Bailit J.L., Gonzalez-Quintero V.H. (2010). Contemporary patterns of spontaneous labor with normal neonatal outcomes. Obstet. Gynecol..

[7] Caughey A.B., Cahill A.G., Guise J.M., Rouse D.J., American College of Obstetricians and Gynecologists, The Society for Maternal–Fetal Medicine (2014). Safe prevention of the primary cesarean delivery. Am. J. Obstet. Gynecol..

[8] ACOG/SMFM (2014). Obstetric care consensus no. 1: Safe prevention of the primary cesarean delivery. Obstet. Gynecol..

[9] Ferber S.G., Granot M., Zimmer E.Z. (2005). Catastrophizing labor pain compromises later maternity adjustments. Am. J. Obstet. Gynecol..

[10] Jones L., Othman M., Dowswell T., Alfirevic Z., Gates S., Newburn M., Jordan S., Lavender T., Neilson J.P. (2012). Pain management for women in labour: An overview of systematic reviews. Cochrane Database Syst. Rev..

[11] Smith L.A., Burns E., Cuthbert A. (2018). Parenteral opioids for maternal pain management in labour. Cochrane Database Syst. Rev..

[12] Konefal H., Jaskot B., Czeszynska M.B. (2012). Pethidine for labor analgesia; monitoring of newborn heart rate, blood pressure and oxygen saturation during the first 24 hours after the delivery. Ginekol. Pol..

[13] Kadirogullari P., Yalcin Bahat P., Sahin B., Gonen I., Seckin K.D. (2021). The Effect of Pethidine Analgesia on Labor Duration and Maternal-Fetal Outcomes. Acta Biomed..

[14] Keskin H.L., Keskin E.A., Avsar A.F., Tabuk M., Caglar G.S. (2003). Pethidine versus tramadol for pain relief during labor. Int. J. Gynaecol. Obstet..

[15] Thomson A.M., Hillier V.F. (1994). A re-evaluation of the effect of pethidine on the length of labour. J. Adv. Nurs..

[16] Ballas S., Toaff M.E., Toaff R. (1976). Effects of intravenous meperidine and meperidine with promethazine on uterine activity and fetal heart rate during labor. Isr. J. Med. Sci..

[17] Sica-Blanco Y., Rozada H., Remedio M.R. (1967). Effect of meperidine on uterine contractility during pregnancy and prelabor. Am. J. Obstet. Gynecol..

[18] Kayacan N., Ertugrul F., Arici G., Karsli B., Akar M., Erman M. (2007). In vitro effects of opioids on pregnant uterine muscle. Adv. Ther..

[19] Zhang Y., Akins M.L., Murari K., Xi J., Li M.J., Luby-Phelps K., Mahendroo M., Li X. (2012). A compact fiber-optic SHG scanning endomicroscope and its application to visualize cervical remodeling during pregnancy. Proc. Natl. Acad. Sci. USA.

[20] Milwidsky A., Finci-Yeheskel Z., Mayer M. (1993). Direct stimulation of urokinase, plasmin, and collagenase by meperidine: A possible mechanism for the ability of meperidine to enhance cervical effacement and dilation. Am. J. Perinatol..

[21] Gomez Slagle H.B., Hoffman M.K., Fonge Y.N., Caplan R., Sciscione A.C. (2022). Incremental risk of clinical chorioamnionitis associated with cervical examination. Am. J. Obstet. Gynecol. MFM.

[22] Doussot M., Barrois M., Anselem O., Tsatsaris V. (2022). Factors Associated with Prolonged Duration of Labor in Medical Termination of Pregnancy in the 2nd and 3rd Trimesters. Gynecol. Obstet. Fertil. Senol..

[23] El-Refaie T.A., El-Said M.M., Shoukry A.A., Khafagy S.M., El-Din A.S., Badawy M.M. (2012). Meperidine for uterine dystocia and its effect on duration of labor and neonatal acid-base status: A randomized clinical trial. J. Obstet. Gynaecol. Res..

[24] Orhan Şahin S.G., Çetinkaya N., Mihmanlı V., Yıldırım G., Tekirdağ A.İ. (2022). Duration of Labor with Meperidine versus Placebo in Singleton Term Pregnancies: A Randomized Placebo Controlled Study. Eur. Arch. Med. Res..

[25] Kamyabi Z., Naderi T., Ramazani A. (2003). A randomized, placebo-controlled trial of the effects of pethidine on labor pain, uterine contractions and infant Apgar score. Ann. Saudi Med..

[26] Rezk M., El-Shamy E.S., Massod A., Dawood R., Habeeb R. (2015). The safety and acceptability of intravenous fentanyl versus intramuscular pethidine for pain relief during labour. Clin. Exp. Obstet. Gynecol..

[27] Sliom C.M. (1970). Analgesia during labour: A comparison between dihydrocodeine and pethidine. S. Afr. Med. J..

[28] Tsui M.H., Ngan Kee W.D., Ng F.F., Lau T.K. (2004). A double blinded randomised placebo-controlled study of intramuscular pethidine for pain relief in the first stage of labour. BJOG.

[29] Letic M. (2003). Inaccuracy in cervical dilatation assessment and the progress of labour monitoring. Med. Hypotheses.

